# Machine learning-assisted chemical design of highly efficient deicers

**DOI:** 10.1038/s41598-024-62942-y

**Published:** 2024-06-07

**Authors:** Kai Ito, Arisa Fukatsu, Kenji Okada, Masahide Takahashi

**Affiliations:** Department of Materials Science, Graduate School of Engineering, Osaka Metropolitan University, 1-1 Gakuen-Cho, Naka-Ku, Sakai, Osaka 599-8531 Japan

**Keywords:** Deicer, Machine learning, Boosting, SHAP, Ice-melting mechanism, Deicing, Materials chemistry, Design, synthesis and processing

## Abstract

The use of deicers in urban areas, on runways and aircrafts has raised concerns about their environmental impact. Understanding the ice-melting mechanism is crucial for developing environmentally friendly deicers, yet it remains challenging. This study employs machine learning to investigate the ice penetration capacity (IPC) of 21 salts and 16 organic solvents as deicers. Relationships between their IPC and various physical properties were analysed using extreme gradient boosting (XGBoost) and Shapley additive explanation (SHAP). Three key ice-melting mechanisms were identified: (1) freezing-point depression, (2) interactions between deicers and H_2_O molecules and (3) infiltration of ions into ice crystals. SHAP analysis revealed different ice-melting factors and mechanisms for salts and organic solvents, suggesting a potential advantage in combining the two. A mixture of propylene glycol (PG) and sodium formate demonstrated superior environmental impact and IPC. The PG and sodium formate mixture exhibited higher IPC when compared to six commercially available deicers, offering promise for sustainable deicing applications. This study provides valuable insights into the ice-melting process and proposes an effective, environmentally friendly deicer that combines the strengths of organic solvents and salts, paving the way for more sustainable practices in deicing.

## Introduction

Deicers are solid or liquid materials that have the dual purpose of melting ice and snow (de-icing) while also prevent ice formation (anti-icing). In cold regions, deicers are essential for maintaining safety and convenience on urban roads, rooftops, airport runways and aircraft wings. Thus, deicers are an essential part of social lives, especially in cold regions, and their environmental impact must be considered, as deicers containing chloride compounds can permeate the soil adjacent to roads, harming plants and causing soil hardening^1^. They can also lead to water pollution and harm aquatic organisms when washed into rivers or ponds from drainage channels. Additionally, they may corrode metals infrastructure^2–7^, vehicles^2,4,8–10^ and other structures. Acetate salts, formate salts and glycols have lower toxicity^11,12^ and minimal corrosive effects^13^, but may still pose risks by consuming significant oxygen during decomposition, potentially harming aquatic life^14,15^. Therefore, developing deicers with minimal environmental impact is critical to address these issues and reduce the overall environmental impact.

In the pursuit of developing deicers that are both environmentally friendly and highly efficient, a key aspect lies in understanding ice-melting mechanisms. Currently, when salt is used as a deicer, solid salts dissolve in a thin water layer (QLL: quasi-liquid layer) on the surface of the ice, forming a solution that lowers the freezing point. Subsequently, the ice melts until the concentration of the salt in the solution is diluted to a point where its freezing point matches the external temperature^16^. In considering ice-melting mechanisms, research has largely focused on freezing point depression. However, Koefod reported that solutions with a high degree of freezing point depression do not necessarily exhibit high de-icing capabilities^17^. Muthumani and Shi also explored the relationship between freezing point depression and de-icing capacity for four agro-based deicers and chloride/inorganic compounds. Their findings indicate that while the salt solution mixed with agro-based deicers did experience freezing point depression, it did not result in improved de-icing capacity^18^. These results emphasise the need for a more detailed understanding of the entire ice-melting mechanism, necessitating the establishment of more comprehensive mechanisms. In this study, machine learning is employed to identify the ice-melting factors of deicers to uncover these mechanisms. The objective is to contribute to the development of deicers with higher ice penetration capacity (IPC) and enhanced functionality.

IPC is a key metric for assessing deicer efficiency. It denotes a deicer’s ability to effectively penetrate and disintegrate ice and snow. Deicers with higher IPC can swiftly and evenly penetrate ice and snow, enabling rapid and widespread dissolution of substantial ice volumes with minimal deicer application. This efficiency can reduce deicer usage, thereby minimizing environmental impact. Systematically understanding IPC holds potential for improving deicer performance and mitigating environmental impact. Therefore, a homemade experimental apparatus (Fig. S1) was used to precisely compare the IPC of deicers. Conventional deicers often involve salts such as chlorides and carboxylates, as well as organic solvents like PG. In the present work, 21 types of salt solutions and 16 types of organic solvents were evaluated according to a reported method^19^ using a homemade experimental apparatus. Additionally, physical properties such as pH and freezing points were measured or obtained from PubChem^20^, accumulating approximately 850 data points for machine learning analysis.

For machine learning, we used extreme gradient boosting (XGBoost)^21^, a type of gradient boosting, algorithm. Machine learning models, including XGBoost, face a notable issue the “black-box problem”–it can be challenging to comprehend how the model generates its output based on the input data. To address this, we focused on Explainable AI (XAI) which pertains to technologies and research areas in machine learning where the processes leading to prediction or estimation results are explainable to humans. In this context, we used SHapley Additive exPlanation (SHAP)^22^, a methodology that assesses the contribution of each variable (feature) to the model's prediction results. SHAP enables visualization of how changes in specific feature variables impact the model’s output.

Considering the discussed ice-melting mechanism and IPC factors determined by SHAP, we anticipated an improvement in IPC by mixing salts with organic solvents. Therefore, we conducted measurements of IPC of these mixtures and found variations—some combinations increased IPC while others decreased it. SHAP analysis helps us understand the parameters that facilitated IPC improvement through interactions between salts and organic solvents.

In the pursuit of developing environmentally friendly deicers, we focused on a mixture of PG, an environmentally conscious choice among organic solvents, and a salt solution that emphasises high IPC. Among various combinations of PG and salt solutions, we identified the mixture with the highest IPC. Opting for the environmentally friendly organic salt, sodium formate, over inorganic salts, we design a novel deicer. Comparing the IPC of the PG and sodium formate mixture with commercially available deicers, we found that our mixture demonstrated higher IPC. Deicers with high IPC can permeate ice widely and evenly, melting substantial amounts of snow and ice efficiently with minimal usage. Consequently, our novel deicer holds potential to reduce deicer usage, simplify de-icing operations, and minimise environmental impact.

## Results and discussion

### Contribution of each explanatory variable to the IPC of salt solutions and organic solvents

**Figure 1:**
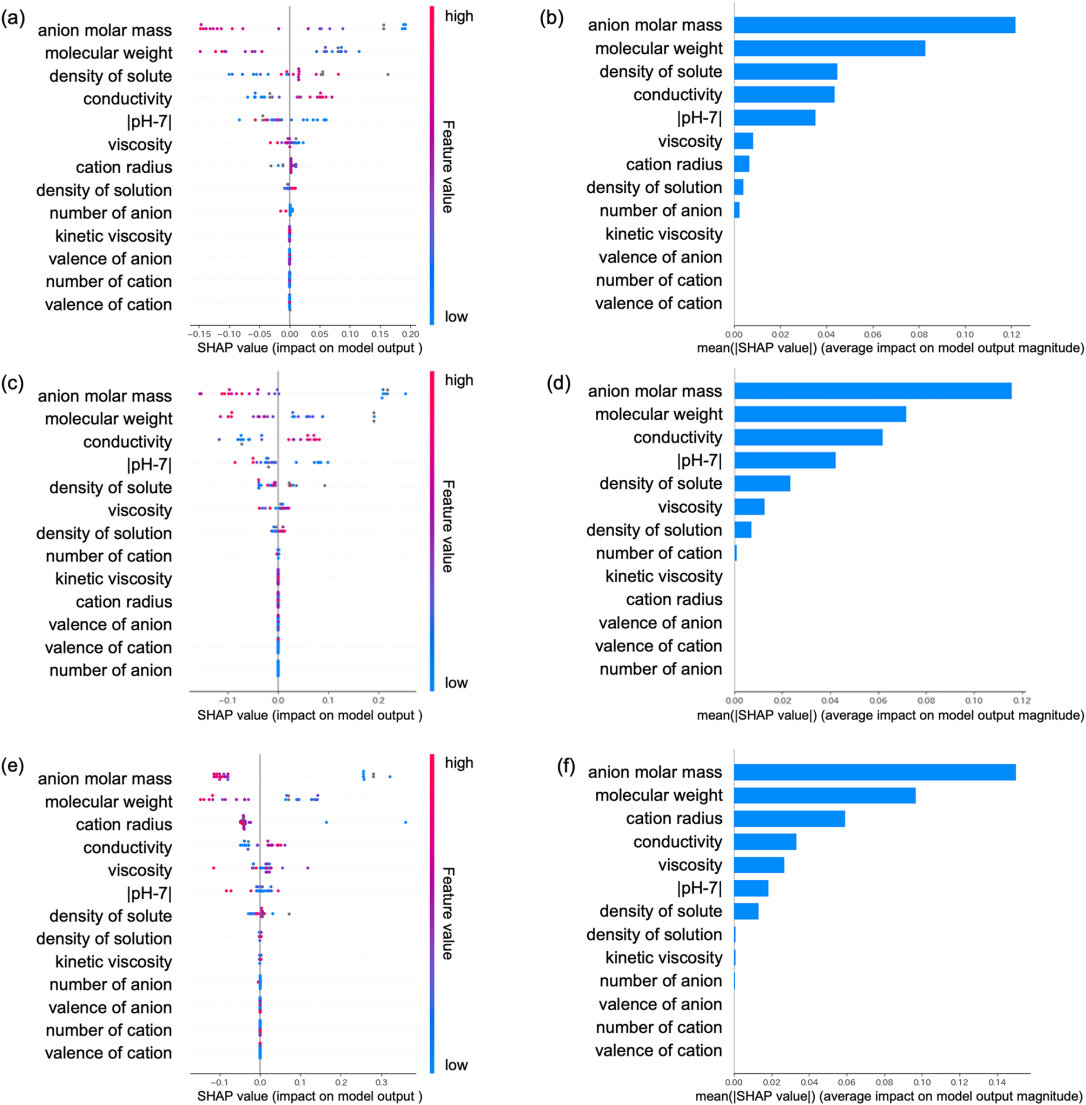
SHAP value of salt solutions at (**a**) 0–5 min, (**c**) 0–10 min and (**e**) 0–30 min, and feature importance at (**b**) 0–5 min, (**d**) 0–10 min and (**f**) 0–30 min.

Figure 1a displays SHAP values for each parameter of the salt solutions over a 0–5-min range of IPC. The vertical axis represents explanatory variables (Table S1), with plot colours indicating the magnitude of each parameter's values: reddish colours indicate high feature values and bluish colours indicate low feature values. The horizontal axis displays the SHAP values, representing the influence of each parameter on the output results. In this context, it represents the influence of each parameter on the IPC of salt solutions in the 0–5-min range. From Fig. 1a, it is evident that variables such as solute density, conductivity and solution density, indicated by reddish plots within the positive SHAP value range, are positively correlated with the 0–5-min IPC. Conversely, variables such as anion molar mass, molecular weight of the whole molecule, |pH-7|, viscosity and the number of anions, indicated by bluish plots within the positive SHAP value range, are negatively correlated with the 0–5-min IPC. In this experiment, |pH-7| was used instead of pH to understand IPC trends concerning solution ionization.

Figure 1b presents the absolute values of SHAP values for each plot shown in Fig. 1a, calculating their averages to represent the feature importance of each parameter for the 0–5-min IPC. Anion molar mass had the highest impact, followed by molecular weight, solute density and conductivity.

Figure 1c illustrates SHAP values for each parameter of the salt solution over the 0–10-min range. In comparison to the correlation observed in the 0–5-min range, solute density and viscosity no longer show a correlation, but other parameters exhibit similar trends. Figure 1d illustrates the feature importance of salt solutions in the 0–10-min range. Anion molar mass and molecular weight continue to have significant impact, with conductivity’s influence increasing, and solute density’s influence decreasing.

Figure 1e demonstrates SHAP values for each parameter of the saltwater solution in the 0–30-min range. Notably, the cation radius, which did not correlate in SHAP diagrams for 0–5 min and 0–10 min, exhibits a negative correlation in the 0–30 min. Figure 1f presents the feature importance of salt solutions in the 0–30-min range. The influence of cation radius becomes more significant compared to the 0–5-min and 0–10-min ranges.

**Figure 2:**
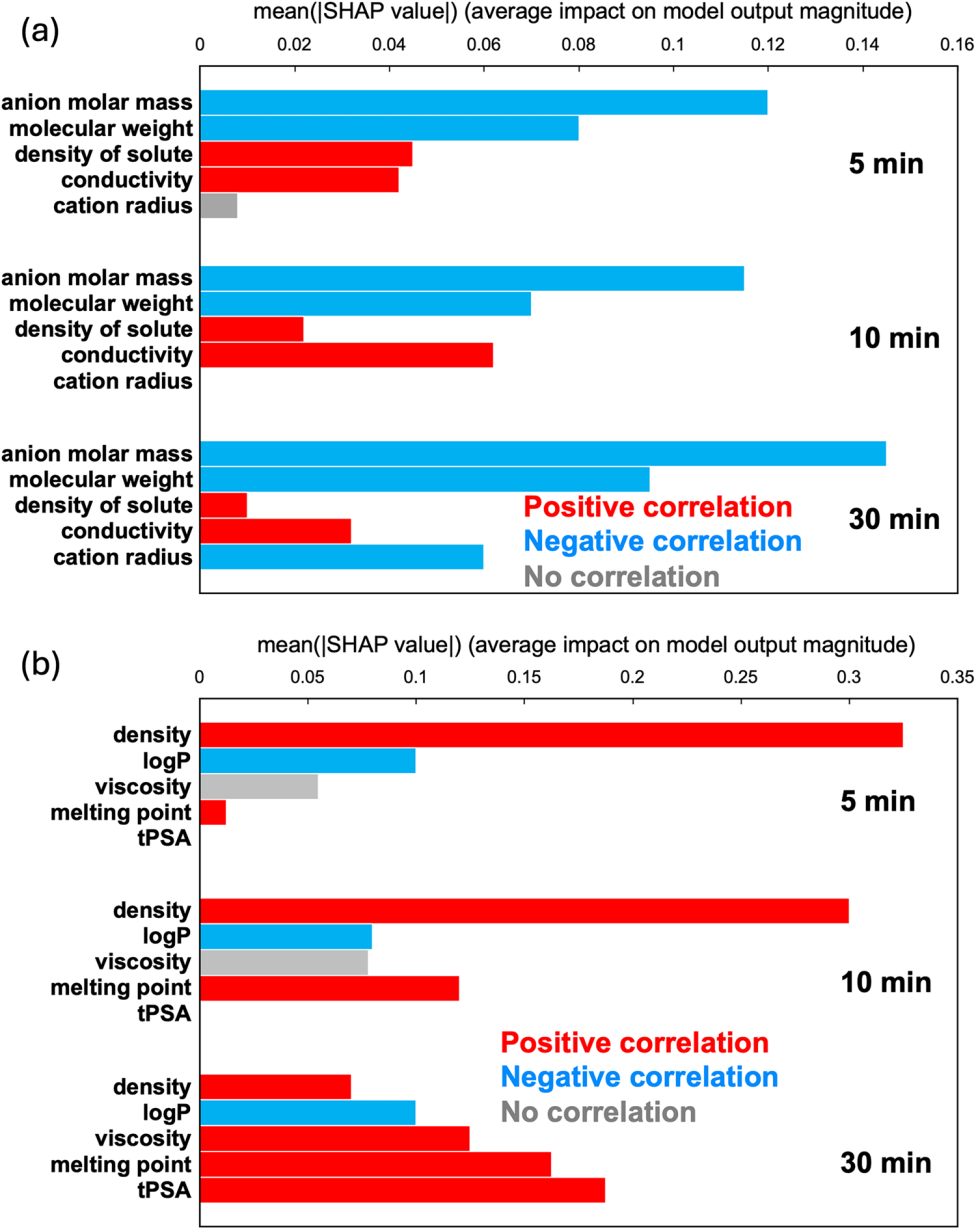
Major feature importance of salt solutions at 0–5, 0–10 and 0–30 min of (**a**) salt solutions and (**b**) organic solvents.

Figure 2a depicts the feature importance of key parameters for salt solutions at 0–5 min, 0–10 min and 0–30 min. Red bars represent positive correlations, while blue bars indicate negative correlations. Across all time intervals, anion molar mass and molecular weight exhibit significant influence, followed by solute density and conductivity. Although the cation radius had a smaller impact at 0–5 min and 0–10 min, it demonstrated significant influence at 0–30 min. It is noteworthy that, while molecular weight shows a negative correlation, using a constant mass of salt implies a positive correlation with the amount of solute material (amount of material = mass/molecular weight). This suggests that a higher number of molecules contributes to improved IPC.

A similar analysis was performed for 16 types of organic solvents, and the contributions of each explanatory variable to IPC were determined (Fig. S2). Figure 2b illustrates the feature importance of key parameters for the 0–5 min, 0–10 min and 0–30 min time intervals. Positive correlations were observed for density, viscosity, melting point and topological polar surface area (tPSA), while octanol/water partition coefficient (logP) displayed a negative correlation. Overall, density had a strong influence at 0–5 min and 0–10 min. In contrast, at 0–30 min, the trend shifted, with tPSA, which had minimal impact at 0–5 min and 0–10 min, showing the most significant influence.

### The ice-melting mechanism derived from SHAP values

SHAP analysis of IPC for salt solutions and organic solvents revealed key parameters influencing the ice-melting process. In salt solutions, molecular weight, anion molar mass, solute density, conductivity and cation radius emerged as crucial factors. The impact of anion molar mass and molecular weight demonstrated significant influence across all time intervals. For organic solvents, density, logP, viscosity, melting point and tPSA were primary parameters. Density had significant influence in the 0–5 min and 0–10 min, while at 0–30 min, tPSA emerged as the most influential factor.

Based on the SHAP analysis results of salt solutions and organic solvents, three major mechanisms for ice melting can be identified.

**Figure 3:**
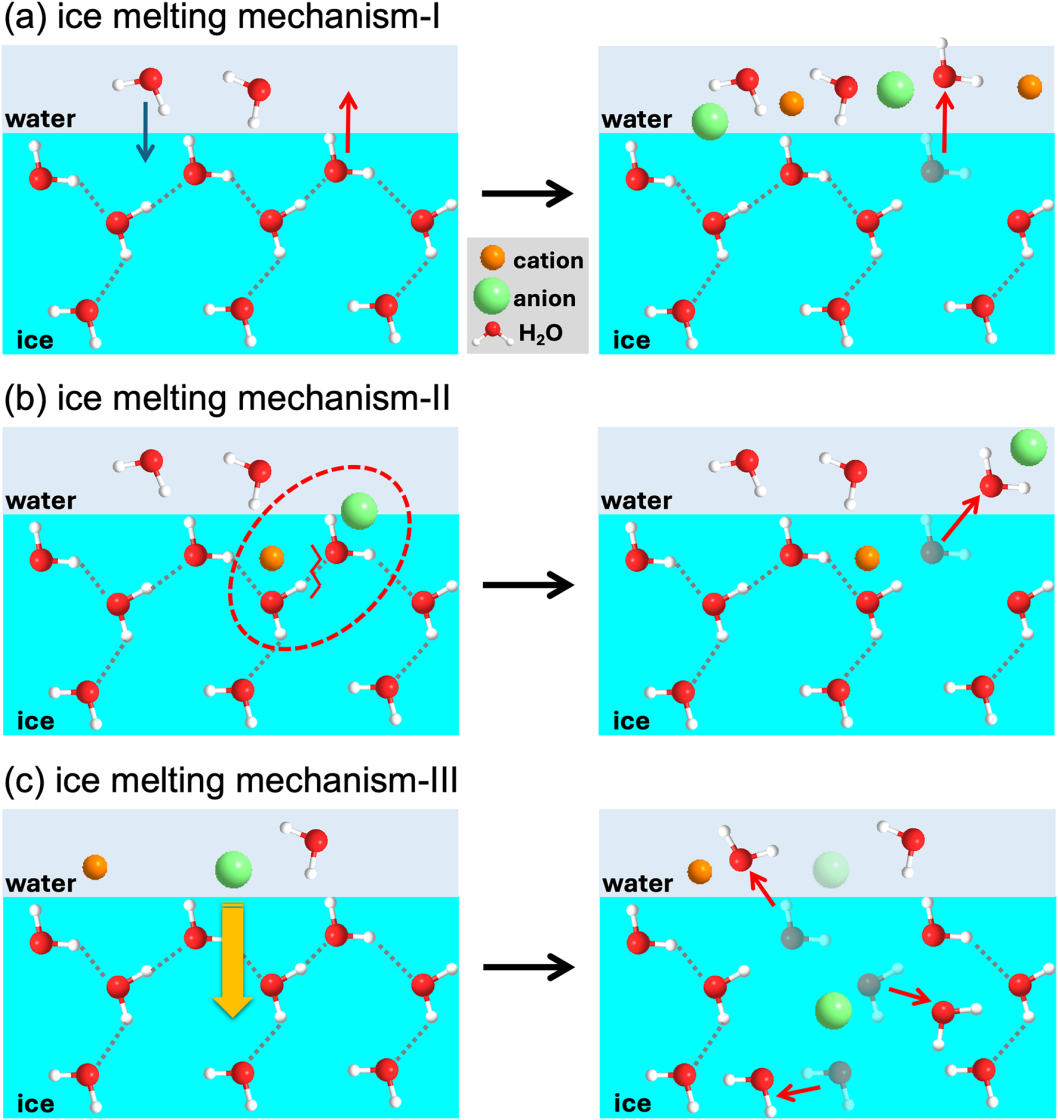
Schematic illustration of the three ice-melting mechanisms; (**a**) freezing point depression (ice melting mechanism-I), (**b**) interaction between deicers and H_2_O molecules (ice melting mechanism-II) and (**c**) infiltration of ions into ice crystals (ice melting mechanism-III).

The first aspect involves ice melting due to freezing-point depression (ice melting I), and can be predicted based on molecular weight. As depicted in the left diagram of Fig. 3a, a thin layer of water (QLL) on ice surface varies in thickness with temperature. In this layer, the number of H_2_O molecules freezing and dissolving from ice crystals are equal, establishing a solid–liquid equilibrium. When a deicer is applied to the ice surface, as shown in the right diagram of Fig. 3a, the presence of deicer in the QLL disrupts the solid–liquid equilibrium between water molecules and ice molecules. The number of dissolved H_2_O molecules surpasses the number of freezing H_2_O molecules. As a result, dissolution of H_2_O molecules progresses, leading to ice melting.

The second mechanism involves ice melting through the interaction between deicer and H_2_O molecules on the ice surface (ice melting II), as illustrated in Fig. 3b. The electrostatic interaction between the positive charge deicer and the negative charge of oxygen atoms of H_2_O molecules, weakens the hydrogen bonds of H_2_O molecules on the ice surface, resulting in melting. This process is influenced by parameters such as conductivity, logP and tPSA. Higher conductivity is associated with enhanced ionization, suggesting increased electrostatic interactions between ions and H_2_O molecules. While, lower logP values indicate hydrophilicity, signifying larger electrostatic interactions between deicer and H_2_O molecules. Furthermore, higher tPSA values correspond to higher polarity, suggesting increased electrostatic interactions between deicer and H_2_O molecules. However, it is believed that the electrostatic interaction between ions and H_2_O molecules in salt solutions is stronger than that between smaller, less polar molecules in organic solvents and H_2_O molecules. Consequently, parameters related to the conductivity of salt solutions are considered to have a more significant impact than parameters representing the polarity of organic solvents.

The third mechanism involves ice melting through the infiltration of ions into ice crystals (ice melting III) as illustrated in Fig. 3c, leads to reorientation of the hydrogen bonding network around H_2_O molecules in the crystallographic sites of ice, particularly influenced by the presence of negative ions. This reorientation promotes defects in the ice crystal structure, leading to disorder and ice melting^23^. Valence and size of both cations and anions contribute to this mechanism. SHAP analysis indicates that cation radius and anion molar mass significantly influence IPC, while ion valence plays a smaller role. Smaller cation radius facilitates ion infiltration, promoting the generation of more crystal defects. Similarly, smaller anion molar mass affects ion size, which also impacts the ease of ion infiltration. This phenomenon is thought to resemble the influence of cation radius, contributing to the facilitation of ion infiltration.

To summarise, in salt solutions, all three ice-melting mechanisms (I, II, III) are considered active. In organic solvents, mechanism-I is primarily operational due to the strength of polarity in mechanism-II.

### Improvement of IPC by mixing salt solutions and organic solvents

Based on Fig. 2, the primary ice-melting factors for salt solutions and organic solvents were identified as solute or solvent density, anion molar mass, molecular weight, conductivity, high polarity and cation radius. These factors were used to discuss the ice-melting mechanism illustrated in Fig. 3. Table 1(a) compares the positive and negative aspects of salt solutions and organic solvents as deicers. Additionally, Table 1(b) summarises the main ice-melting mechanisms and factors over time for salt solutions and organic solvents.

**Table 1:** (a) Positive and negative aspects of salt solutions and organic solvents as deicers, and (b) major ice melting factor s and mechanism s at each time period.

(a)	Salt solutions	Organic solvents		
+	I. Freezing-point depression II. Interaction between deicers and H_2_O molecule III. Infiltration of ions into ice crystals	Molecular concentration		
−	Solubility	I. Freezing-point depression		

(b)	Salt solutions		Organic solvents	
	Ice melting factors	Ice melting mechanism	Ice melting factors	Ice melting mechanism
5 min	Molecular weight	I	Density	I
	Conductivity	II		
10 min	Molecular weight	I	Density	I
	Conductivity	II		
30 min	Molecular weight	I	Density	I
	Conductivity	II	Polarity	(II)
	Cation radius	III		

From Table 1(b), salt solutions demonstrate ice-melting mechanisms-I and -II at 5, 10 and 30 min, with the addition of mechanism-III at 30 min. Organic solvents, in contrast, primarily operate under mechanism-I for all observed durations, reflecting a key difference in the variety of ice-melting mechanisms between salt solutions and organic solvents.

Next, the parameter of the number of molecules, which significantly influences ice-melting mechanism-I, is considered. In salt solutions, where water serves as a solvent within the deicer, the number of molecules of solute molecules is limited. Therefore, the number non-H_2_O molecules per unit volume in the deicer is relatively low. Conversely, organic solvents consist solely of the molecules, resulting in a higher number of non-H_2_O molecules per unit volume. To sustain and effectively operate ice-melting mechanism-I continuously, a significant number of molecules in the ice/QLL interfaces is necessary. Organic solvents, with a higher concentration of non-H_2_O molecules per unit volume, excel as deicers for maintaining the continuous and effective operation of ice-melting mechanism-I.

In summary, salt solutions offer the advantage of utilizing three different ice-melting mechanisms, but their solubility limitations result in fewer molecules, in other words, lower concentration. On the other hand, the positive aspect of organic solvents as deicer lies in their abundance of molecules, while the disadvantage is having only one primary ice-melting mechanism. Mixing these components to create a deicer may complement each other's weaknesses and improve IPC.

### Selection of salt solutions and organic solvents

From 16 salt solutions and 21 organic solvent solutions, five samples each were selected based on factors such as IPC, material types (functional groups or types of anions) and environmental impact. Salt solutions selected included LiCl with the highest IPC, NaCl, which is cost-effectiveness and common use as a deicer, HCOONa as an environmentally friendly organic salt with high IPC, MgCl_2_ for its divalent cation and high IPC, and NaBr for comparison based on anion differences. Organic solvents were chosen based on high IPC, including formamide with the highest IPC, formic acid with a carboxyl group, ethylene glycol with two hydroxyl groups, dimethyl sulfoxide with a sulfo group and PG, which is relatively environmentally friendly and already used as an existing deicer. The IPC of a total of 25 mixed solutions (5 × 5) was measured. The IPC of each mixture of salt solution and organic solvent was compared to the average IPC of its constituents (average IPC = ((IPC of a salt solution) + (IPC of an organic solvent))/2), observing changes in IPC due to mixing.

### Comparison of mixtures of salt solutions and organic solvents

**Figure 4:**
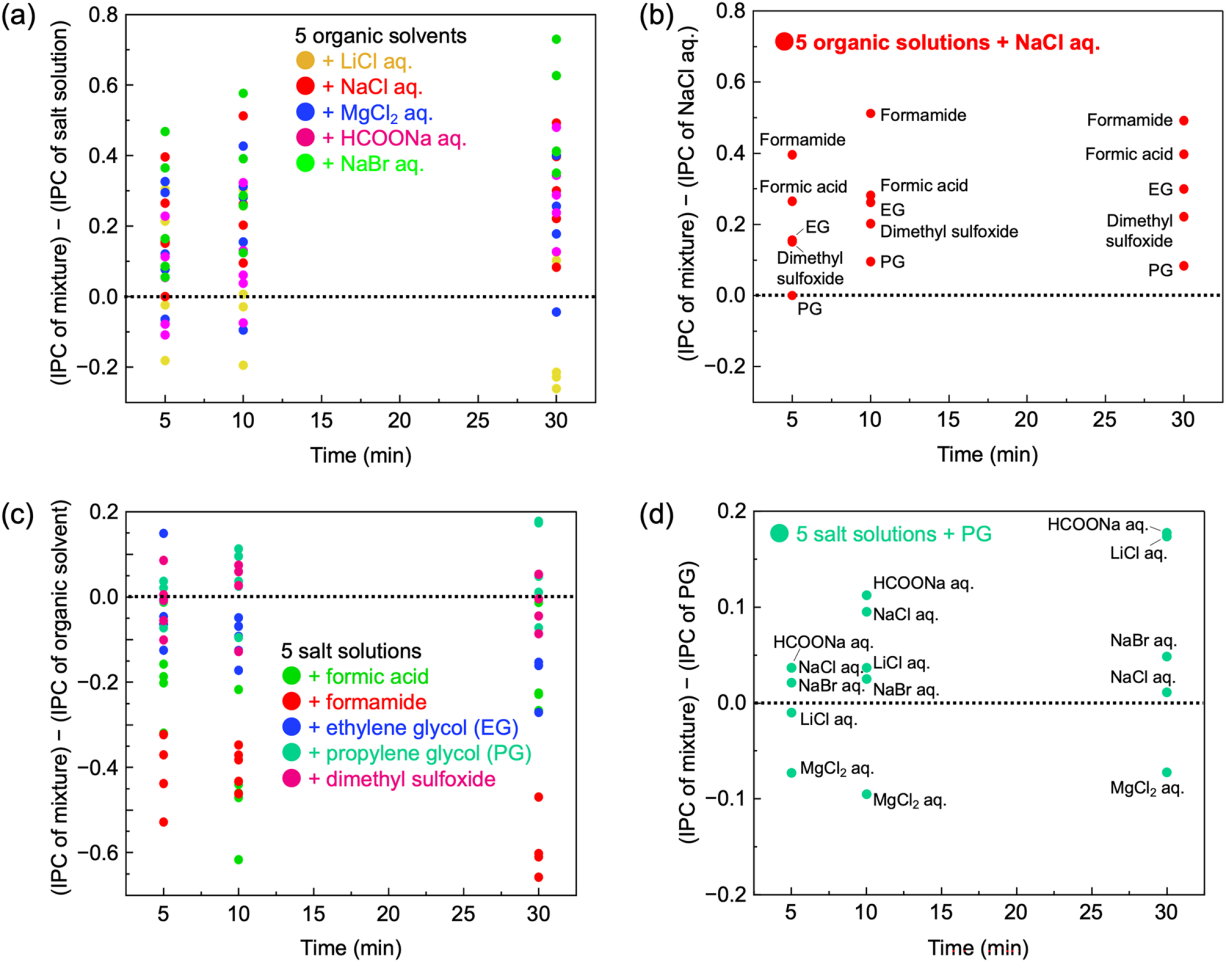
Comparison of IPC between mixtures for (**a**) five salt solutions, (**b**) NaCl aq., (**c**) five organic solvents and (**d**) PG.

Figure 4a shows the difference in IPC between mixtures of salt solutions and organic solvents compared to individual salt solutions. Mixtures containing LiCl generally exhibit lower IPC compared to LiCl alone, while mixtures with other salts showed improved IPC. Figure 4b highlights mixtures of NaCl with the five organic solvents, indicating that the IPC of NaCl mixtures with organic solvents follows a similar order of increasing IPC observed in the plot of individual organic solvents presented in Fig. S3.

Figure 4c compares IPC difference between mixtures of salt solutions and organic solvents and the individual organic solvents. Results show that, at all-time intervals, individual organic solvents generally have higher IPC. For instance, mixture containing formamide and salt solution had lower IPC than formamide alone. However, mixtures of PG and dimethyl sulfoxide with salt solutions showed increase in IPC, suggesting that the difference in IPC between mixtures and individual solvents depends on the IPC magnitude of the individual solvent. Formamide showed a larger negative difference, while PG and dimethyl sulfoxide exhibited predominantly positive values. Figure 4d showcases excerpts from Fig. 4c featuring plots of mixtures of PG and salt solutions. Overall, mixtures of PG with salt solutions had higher IPC compared to PG alone (Fig. 4d). Unlike NaCl mixtures, whose IPC mainly depends on the IPC of the individual organic solvent (as shown in Fig. 4b), the IPC of PG and salt solution mixtures does not solely rely on the IPC of the salt solution alone. These results suggest the presence of synergistic parameters that contribute to the increase in IPC between PG and salt solutions.

### The cause of the difference in IPC by mixing salt solutions and organic solvents

**Figure 5:**
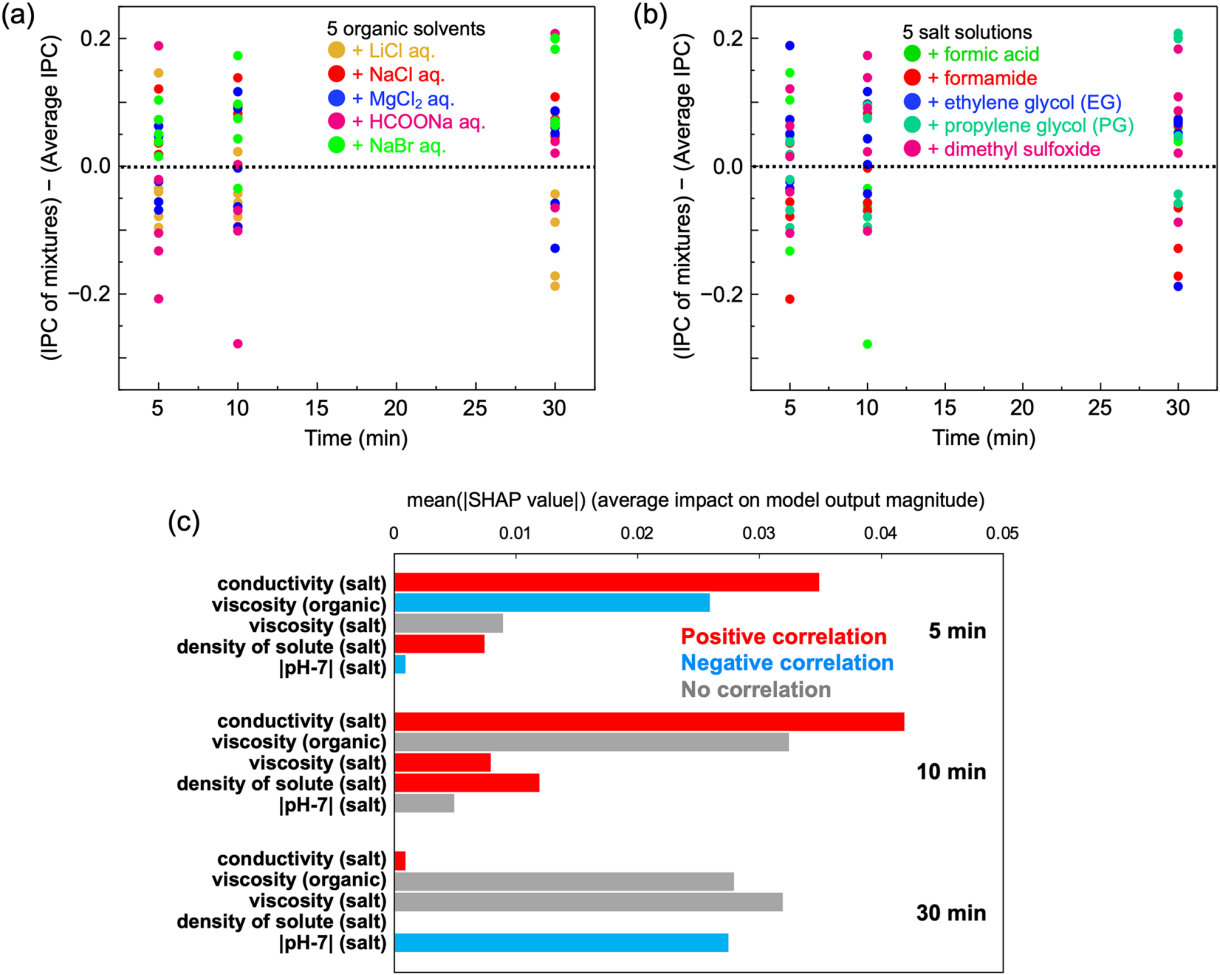
Comparison of IPC between the mixture and the average of each component. The data is colour-coded based on (**a**) salt solutions, (**b**) organic solvents and (**c**) feature importance of the parameters indicating the causes of changes in IPC.

To investigate the interaction between salt solutions and organic solvents, IPC of mixtures was compared with the average IPC of each component. SHAP was used to analyse the causes of the difference in IPC. Figure 5a,b are similar plots, where Fig. 5a is color-coded based on salt solutions, while Fig. 5b is color-coded based on organic solvents. From Fig. 5a, mixtures with NaBr or NaCl exhibit a larger degree of increased IPC. Figure 5b shows that mixtures with dimethyl sulfoxide, ethylene glycol or formic acid also demonstrate a higher increase in IPC.

Figure 5c presents mean SHAP values calculated using the difference in IPC between the mixture and the average of each component as the target variable, and the physical properties of the components as explanatory variables. It confirms that physical properties of salt solutions, such as conductivity, solute density and |pH-7| as well as the physical properties of organic solvents such as viscosity, influence the change in IPC of the mixture.

**Figure 6:**
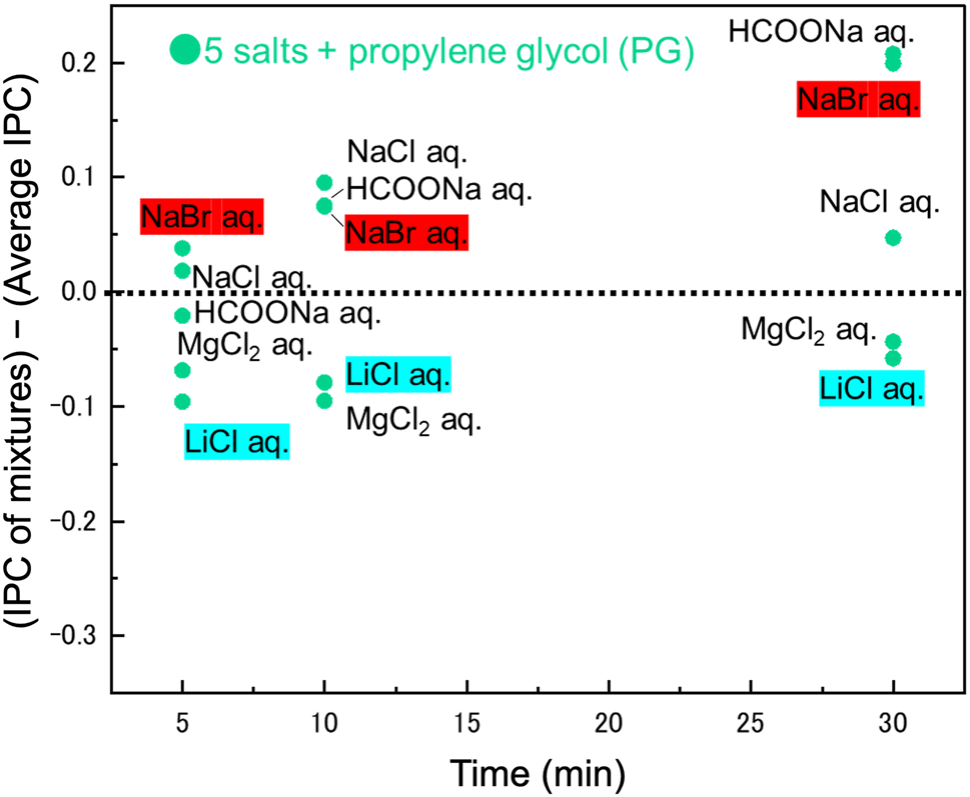
Comparison between IPC of mixture and individual average IPC for PG.

Figure 6 displays extracted data of the mixtures containing PG from Fig. 5a,b. Samples labelled as PG + NaBr (in red) show an increase in IPC due to mixing, while those labelled as LiCl (in blue) indicate a decrease in IPC. Figure 5 identifies the factors influencing the change in IPC when mixing salt solution and organic solvent. In the case of salt solutions, factors such as conductivity, solute density and |pH-7| were primarily considered (as listed in Table S2(a)). Comparing these parameters, NaBr exhibited higher values for both conductivity and solute density, and a lower |pH-7| than LiCl. This observation aligns with the trends shown in Fig. 5, where an increase in IPC is demonstrated. Mixing PG, an organic solvent that primarily exhibits mechanism-I of ice penetration, with salt solutions, brings the additional effects of mechanisms-II, III into play. Table S2(b) reveals that mixing salt solutions with PG enhances conductivity, promoting IPC through interactions between ice molecules and deicers, as described in mechanism-II. Furthermore, since salt solutions contain ions, ion infiltration into ice crystals, as described in mechanism-III, is presumed to occur simultaneously.

### Comparing the proposed deicer with commercial deicers

PG, commonly used as a deicer, was mixed with various salt solutions to improve IPC and compared to the IPC of commercial deicers. The commercial deicers used in the study included KILFROST DF Plus Type I (PG-based, for aircraft deicing), Clearway SF3 (sodium formate-based, for runway deicing), Tocus Si (potassium silicate-based, for urban deicing), Snow Tokesuko (calcium chloride-based, for snow melting), Safe Even When Frozen, salt-free type (urea-based, for anti-icing) and Glaco (ethanol-based, for window deicing), which were all purchased from the market. Clearway SF3, Snow Tokesuko and Safe Even When Frozen, originally intended for use as solids, were tested as 25w/w% aqueous solutions in this study.

**Figure 7:**
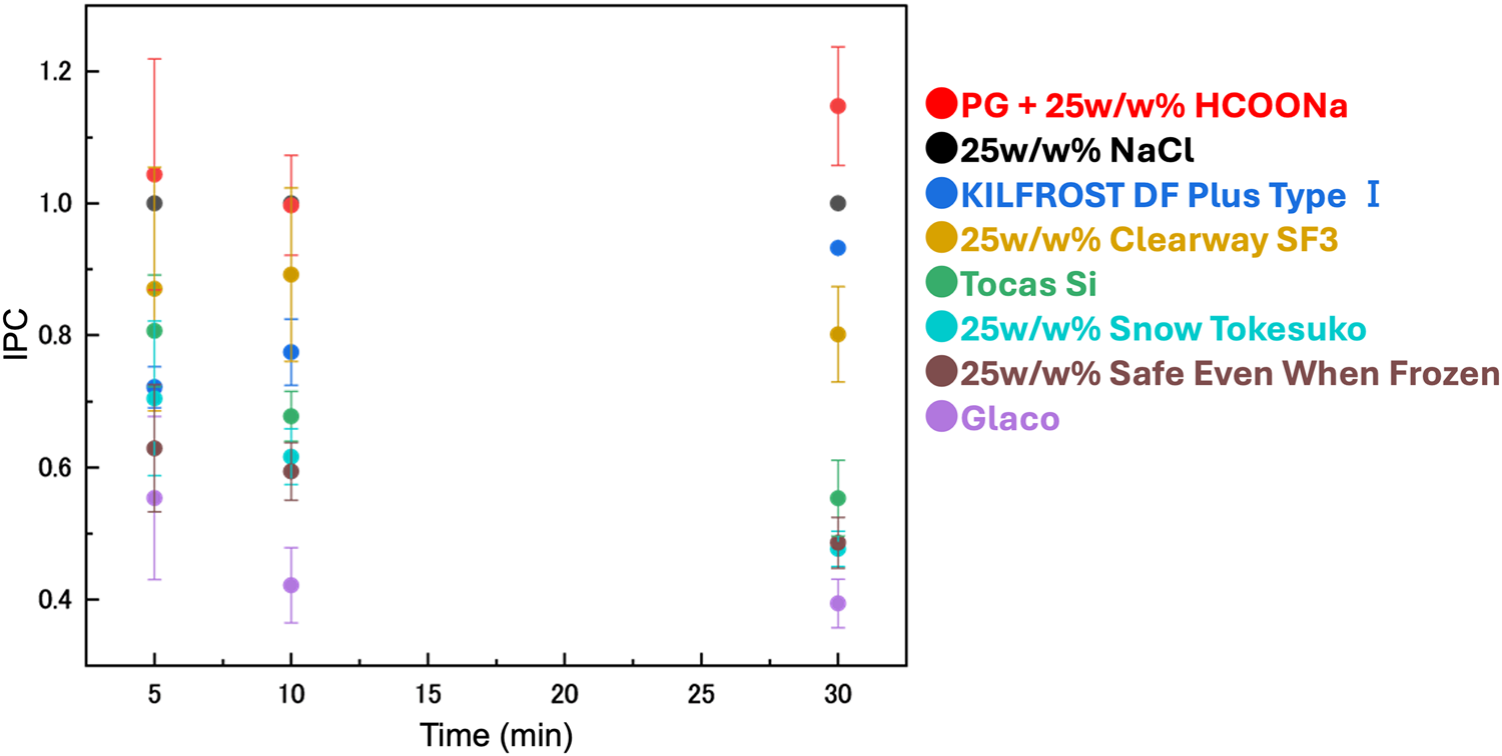
Comparison between the developed deicer and commercial deicers.

Figure 7 shows the average IPC results over four ice penetration tests for each deicer. The mixture of PG and HCOONa displayed a higher IPC compared to other existing deicers across all time intervals of 5, 10 and 30 min. This performance is attributed to synergistic effect of combining the salt solution and organic solvent. The environmental impacts of the deicers are summarised in Table S3. The mixture of PG and HCOONa exhibits lower corrosive effects compared to typical salt-based deicers, and demonstrates reduced chemical oxygen demand (COD) and biochemical oxygen demand (BOD) compared to organic-based deicers. These characteristicts suggest that mixtures of PG and sodium formate are effective deicing agents for roads and runways due to their low toxicity^11,12^ and minimal corrosive effects^13^.

## Conclusion

In the salt solutions, anion molar mass significantly impacted IPC across all time intervals, followed by the influence of molecular weight. Solute and conductivity also played influential roles. Moreover, cation radius was observed to influence the results only at 30 min. For organic solvents, solvent density had a strong influence at 5 and 10 min, followed by logP and melting point. Conversely, at 30 min, the trend shifted, with tPSA having the most prominent influence, followed by melting point, viscosity, logP and density. The ice-melting mechanism was examined based on the ice-melting factors derived from SHAP analysis of salt solutions and organic solvents: I. freezing point depression, II. interaction between the deicer and H_2_O molecules and III. infiltration of ions into ice crystals. Organic solvents primarily undergo ice melting through mechanism-I, while salt solutions involved the combined action of mechanisms I, II and III.

Improvement in IPC was achieved by mixing salt solutions and organic solvents. SHAP analysis of revealed significant influences from conductivity, solute density and |pH-7| for salt solutions, while viscosity played a substantial role for organic solvents.

A mixture of PG and HCOONa, characterised by high IPC and low environmental impact, was compared to commercial deicers and found to outperform them. This demonstrated the potential of creating a potent deicer by mixing salt solutions and organic solvents. As a result, a deicer with a higher IPC than the commercial product was successfully developed. The development of such a deicer with large IPC allows for the reduction of deicing agent usage, thereby decreasing environmental impact. Moreover, mixing salt solutions and organic solvents include can lower the concentrations of both components in the deicer, further reducing environmental impact. This approach presents an effective strategy for creating powerful yet eco-friendly deicers.

## Methods

**Figure 8:**
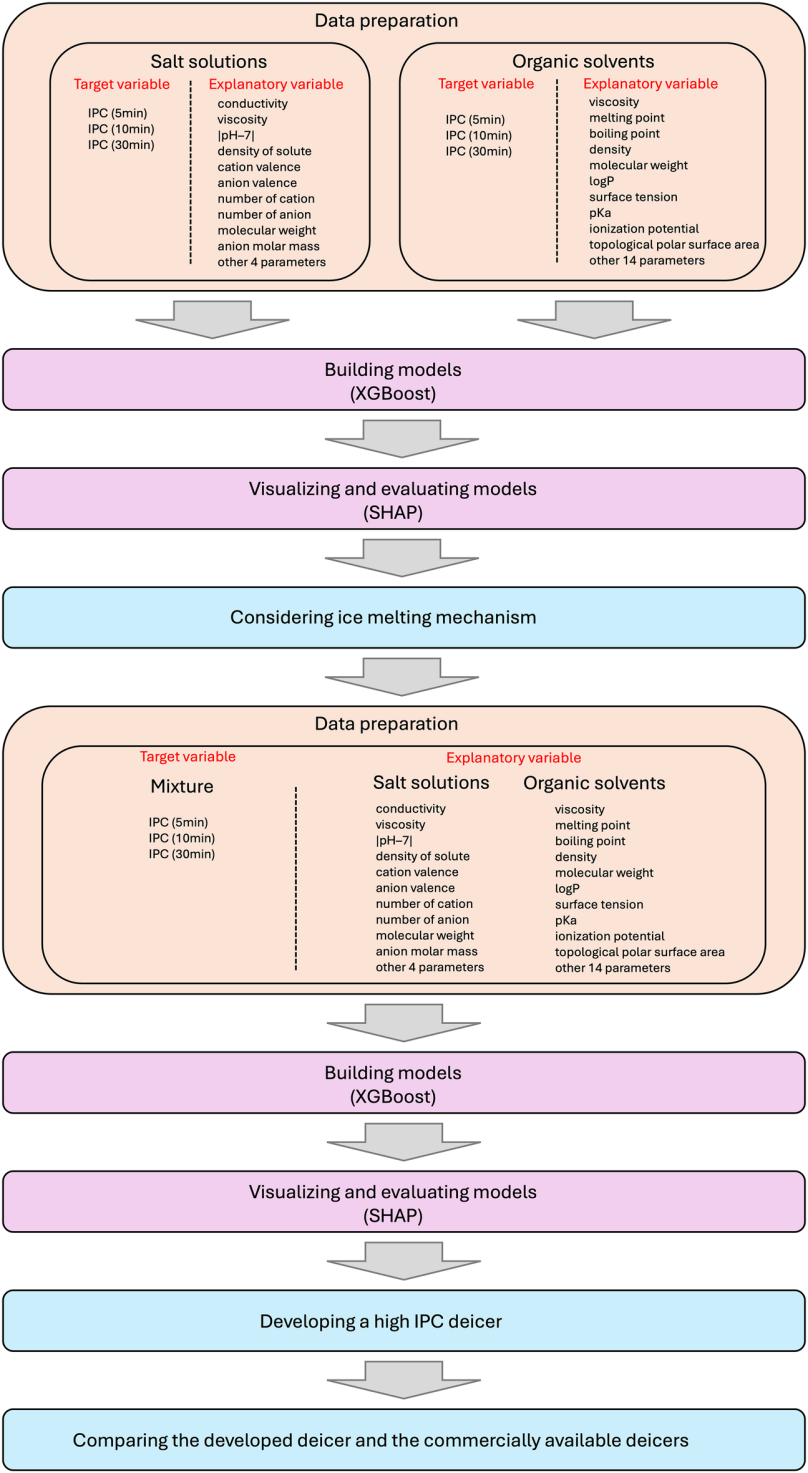
Flowchart of the machine learning process in this study.

The methodology used in this study is summarised in Fig. 8. XGBoost^21^ was employed to model the IPC of deicers, and SHAP^22^ was used to interpret the model’s output. For more information on XGBoost and SHAP, refer to the papers by Chen and Guestrin^21^ and by Lundberg and Lee^22^, respectively.

### Preparation of experimental data

The analysis used samples of 21 types of salt solutions and 16 types of organic solvents for machine learning. The target variables for the machine learning analysis were the IPC values at 0–5 min, 0–10 min, and 0–30 min for both salt solutions and organic solvents. As explanatory variables, 14 physical properties were employed for salt solutions, such as the number of cations and pH, as detailed in Table S1. Additionally, for organic solvents, 24 physical properties were considered, including logP and tPSA. Here, logP represents the octanol/water partition coefficient, indicating water solubility (with lower values representing higher solubility). tPSA refers to the topological polar surface area, representing the area of a molecule's surface that is polar, with higher values suggesting a larger polar surface area.

### Preparation of ice

A glass tube with a diameter of 6 mm and a length of 40 mm was filled with 0.2 mL of distilled water. The tube was then chilled in a refrigerator at − 20 °C for more than 10 h.

### Ice penetration test

The ice penetration test was conducted using a modified method from a report published by the Society of Automotive Engineers, Inc. (SAE International)^19^. The experiment was performed using a home-built apparatus, illustrated in the Supplementary Information (Fig. S1). An antifreeze solution (a 50/50 mixture of ethylene glycol and water) was prepared using a cooling pipe connected to a chiller set at − 5 °C to − 3 °C. The stirrer was placed directly above the cooling tube and adjusted to a speed of approximately 800–1200 rpm (ensuring that the water remained stable and that there was no temperature difference across the left and right sides). The temperature of the antifreeze solution was maintained at − 3.0 °C to − 2.5 °C. A ruler and the glass tube containing 0.2 mL of ice were placed on the antifreeze solution using foam polystyrene for flotation. The system was allowed to equilibrate for 1 h. A standard material 25w/w% NaCl aqueous solution and measurement samples were dispensed in 25 μL volumes into separate glass tubes. The ice penetration test was recorded with a camera, and the lengths of the deicer were measured at 5, 10 and 30 min after the sample solution was dispensed, using a ruler to measure from the underside of the meniscus to the upper surface of the ice.

### Analysis method of ice penetration test

The IPC was determined using a 25% NaCl aqueous solution as the standard material. The IPC for each sample was calculated using the following equation (Eq. 1), where the IPC of the 25% NaCl aqueous solution is normalized to 1.

1 $$IPC = \frac{ice \;penetration\;length\;of\;measurement\;sample}{{ice\;penetration\;length\;of\;25w/w\% \;NaCl \,aq.}}$$

This equation allows for standardised comparison of IPC across different samples. The predicted IPC using machine learning was compared with experimental data and summarised in Table S4 and Fig. S4. Since the model reproduced the raw data, the model was deemed reliable and appropriate for use in this study.

### Supplementary Information

Supplementary Information.

## Data Availability

The datasets used in this study are available from the corresponding authors upon reasonable request.

## References

[1] Fay L, Shi X (2012). Environmental impacts of chemicals for snow and ice control: State of the knowledge. Water Air Soil Pollut..

[2] Shi X, Fay L, Yang Z, Nguyen TA, Liu Y (2009). Corrosion of deicers to metals in transportation infrastructure: Introduction and recent developments. Corros. Rev..

[3] Shi, X., Liu, Y., Mooney, M., Berry, M., Hubbard, B., Fay, L. & Leonard, A. B. Effect of chloride-based deicers on reinforced concrete structures, *Report No. WA-RD 741.1. Washington State Department of Transportation (WSDOT)*, (2010).

[4] Sajid HU, Kiran R, Qi X, Bajwa DS, Battocchi D (2020). Effect of agro-derived corrosion inhibitors on the properties of Portland cement mortar. Constr. Build. Mater..

[5] Vega-Zamanillo Á, Juli-Gándara L, Calzada-Pérez MÁ, Teijón-López-Zuazo E (2020). Impact of temperature changes and freeze—thaw cycles on the behaviour of asphalt concrete submerged in water with sodium chloride. Appl. Sci..

[6] Xie N, Shi X, Zhang Y (2017). Impacts of potassium acetate and sodium-chloride deicers on concrete. J. Mater. Civ. Eng..

[7] Shi X, Fortune K, Smithlin R, Akin M, Fay L (2013). Exploring the performance and corrosivity of chloride deicer solutions: Laboratory investigation and quantitative modeling. Cold Reg. Sci. Technol..

[8] Honarvar Nazari, M. & Shi, X. in *Sustainable Winter Road Operations* (eds Shi, X. &Fu, L.), 241–272 (Wiley, 2018).

[9] Honarvar Nazari, M., Bergner, D. & Shi, X. Managing metallic corrosion on winter maintenance equipment assets. *Environ. Sustain. Transp. Infrastruct.* 61–76 (2015).

[10] Li Y, Fang Y, Seeley N, Jungwirth S, Jackson E, Shi X (2013). Corrosion by chloride deicers on highway maintenance equipment: Renewed perspective and laboratory investigation. Transp. Res. Rec..

[11] Bang SS, Johnston D (1998). Environmental effects of sodium acetate/formate deicer, ice shear. Arch. Environ. Contam. Toxicol..

[12] Lewis AS, Boomhower SR, Marsh CM, Jack MM (2024). Considerations for deriving a safe intake of propylene glycol. Food Chem. Toxicity.

[13] Ropital F (2009). Current and future corrosion challenges for a reliable and sustainable development of the chemical, refinery, and petrochemical industries. Mater. Corros..

[14] Fay L, Shi X (2012). Environmental impacts of chemicals for snow and ice control: State of the knowledge. Water Air Soil Pollut.

[15] Ramakrishna DM, Viraraghavan T (2005). Environmental impact of chemical deicers: A review. Water Air Soil Pollut..

[16] Nilssen K, Klein-Paste A, Wåhlin J (2016). Accuracy of ice melting capacity tests: Review of melting data for sodium chloride. Transp. Res. Rec..

[17] Koefod S (2008). Relationship of eutectic, freezing point, and ice melting capacity in liquid deicers. Transp. Res. Circ..

[18] Muthumani A, Shi X (2017). Effectiveness of liquid agricultural by-products and solid complex chlorides for snow and ice control. J. Cold Reg. Eng..

[19] SAE INTERNATIONAL, AEROSPACE INFORMATION REPORT, AIR6211 (2017).

[20] https://pubchem.ncbi.nlm.nih.gov/

[21] Chen T, Guestrin C (2016). XGBoost: A scalable tree boosting system. KDD.

[22] Lundberg, S. & Lee, S. A unified approach to interpreting model predictions. In *NIPS'17: Proceedings of the 31st International Conference on Neural Information Processing Systems*, 4768–4777 (2017).

[23] Berrens ML, Bononi FC, Donadio D (2022). Effect of sodium chloride adsorption on the surface premelting of ice. Phys. Chem. Chem. Phys..

