# Supplementary material for: Machine learning-assisted chemical design of highly efficient deicers
Source: Sci Rep. 2024 Jun 7;14:12453. doi: 10.1038/s41598-024-62942-y (PMC11161627; doi:10.1038/s41598-024-62942-y)
Supplement: Supplementary file 1 — Supplementary Information. [file 41598_2024_62942_MOESM1_ESM.docx]

**Supplementary Information**

**Machine learning-assisted chemical design of highly efficient deicers**

Kai Ito^1^, Arisa Fukatsu^1*^, Kenji Okada^1^ and Masahide Takahashi^1*^

^1^Department of Materials Science, Graduate School of Engineering, Osaka Metropolitan University, 1-1 Gakuen-cho, Naka-ku, Sakai, Osaka 599-8531, Japan.
E-mail: fukatsu@omu.ac.jp (A.F.), masa@omu.ac.jp (M.T.)

**Figure S1** Homemade experimental apparatus for ice penetration test.

**Table S1** Explanatory variables used in the machine learning analysis.

**Figure S2** SHAP values of organic solvents.

**Figure S3** IPC of pure materials.

**Table S2** Conductivity, solute density and |pH-7| of NaBr aq. and LiCl aq. and conductivity of the mixture of PG and salt solutions.

**Table S3** Environmental impact of deicers investigated in this study.

**Table S4** R-squared values for the predicted data using SHAP and the raw data of IPC.

**Figure S4** The relationship between the predicted data using SHAP and the raw data of IPC.


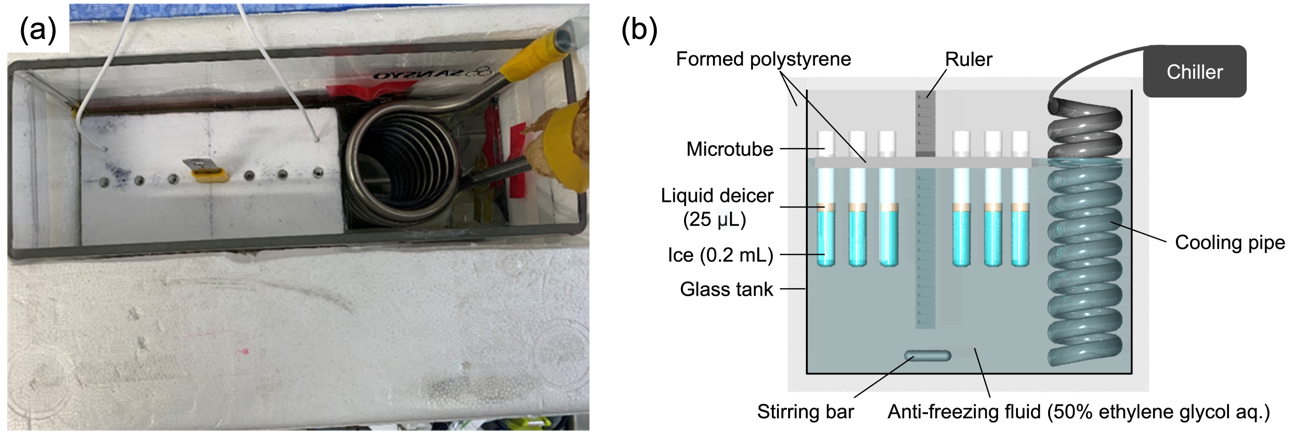


**Figure S1 (a) Photograph and (b) schematic diagram of the home-built experimental apparatus for the ice penetration test.** A glass tank was filled with a 1:1 solution of ethylene glycol and water as an antifreeze solution. A cooling tube connected to a chiller was inserted to cool the solution (the chiller temperature was set to −5 to −3 °C to maintain the antifreeze solution between −3.5 to −2.5 °C). A ruler and a glass tube containing ice were floated on the antifreeze solution using foam polystyrene for support. A deicer was then dispensed into the glass tube, and its IPC was measured by recording the length using the ruler.

**Table S1 Explanatory variables used in the machine learning analysis of (a) salt solutions and (b) organic solvents.** For salt solutions pH, conductivity, viscosity, and density of solution were measured using experimental apparatus, while other parameters were sourced from a chemical database^1^. Dynamic viscosity was calculated utilizing the measured viscosity and the density obtained from the database. In the case of organic solvents, all parameters were sourced from the chemical database.


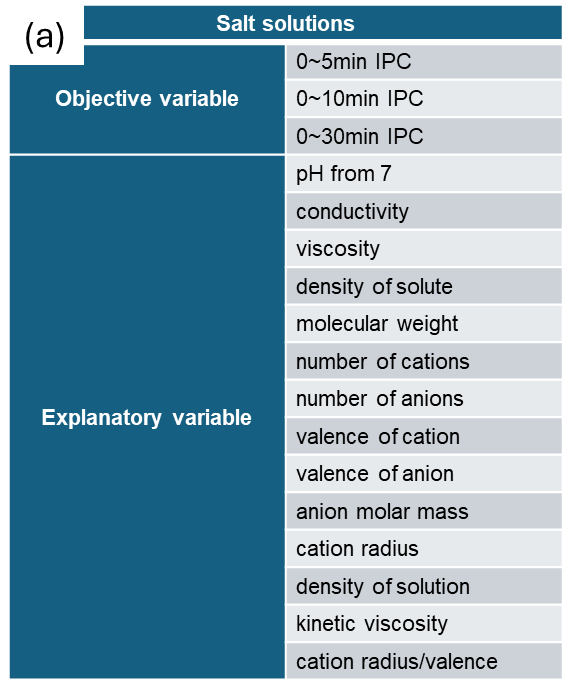


**
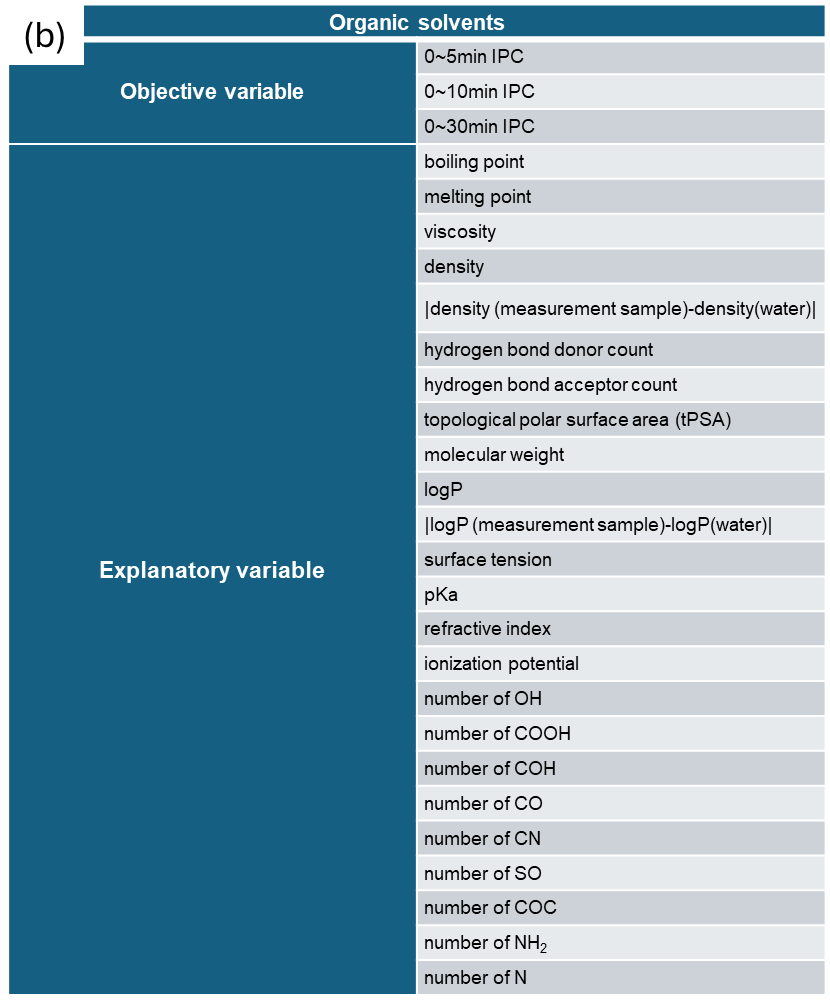
**


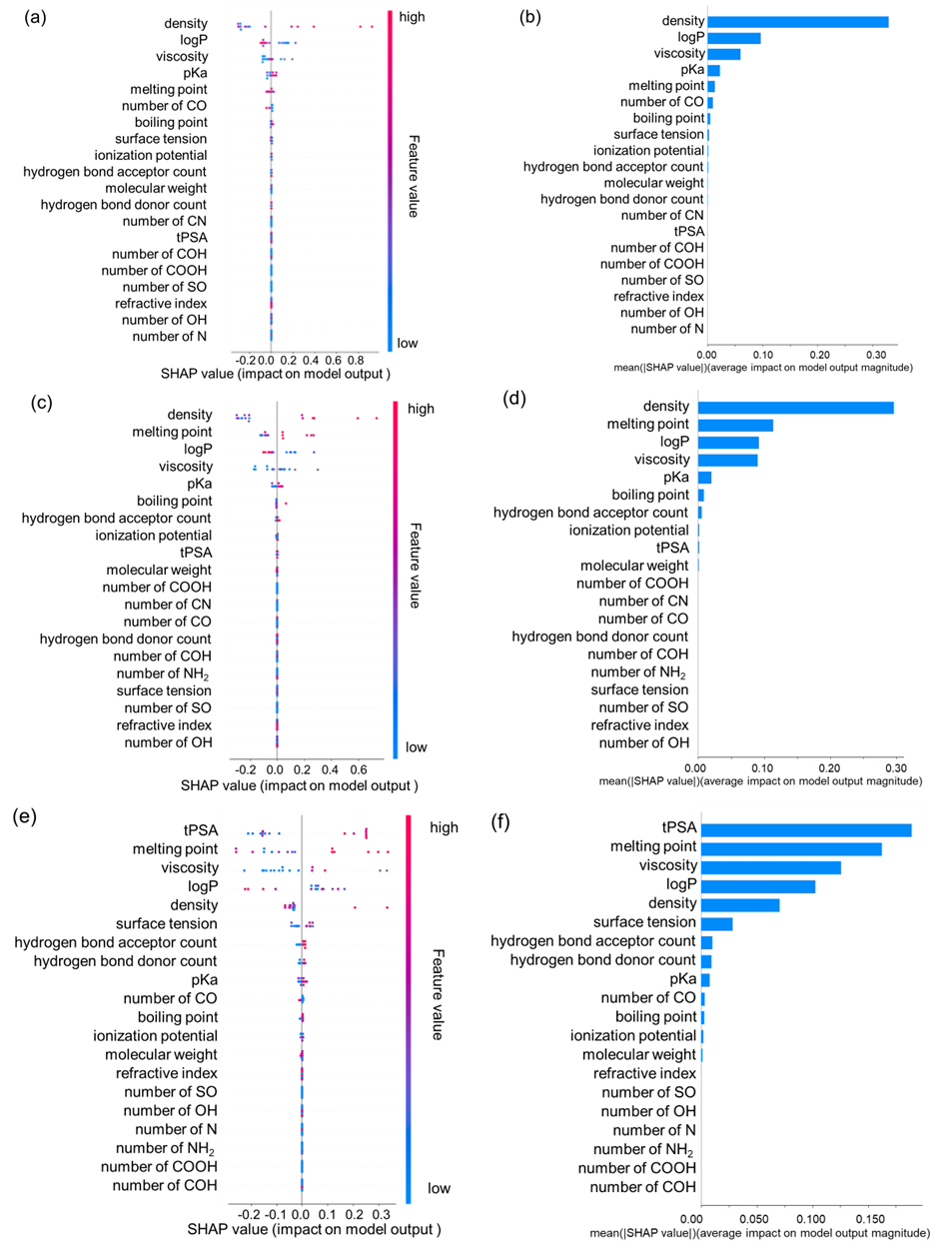


**Figure S2 SHAP values of organic solvents at (a) 0–5 min, (c) 0–10 min and (e) 0–30 min and**

**feature importance at (b) 0–5 min, (d) 0–10 min and (f) 0–30 min.**


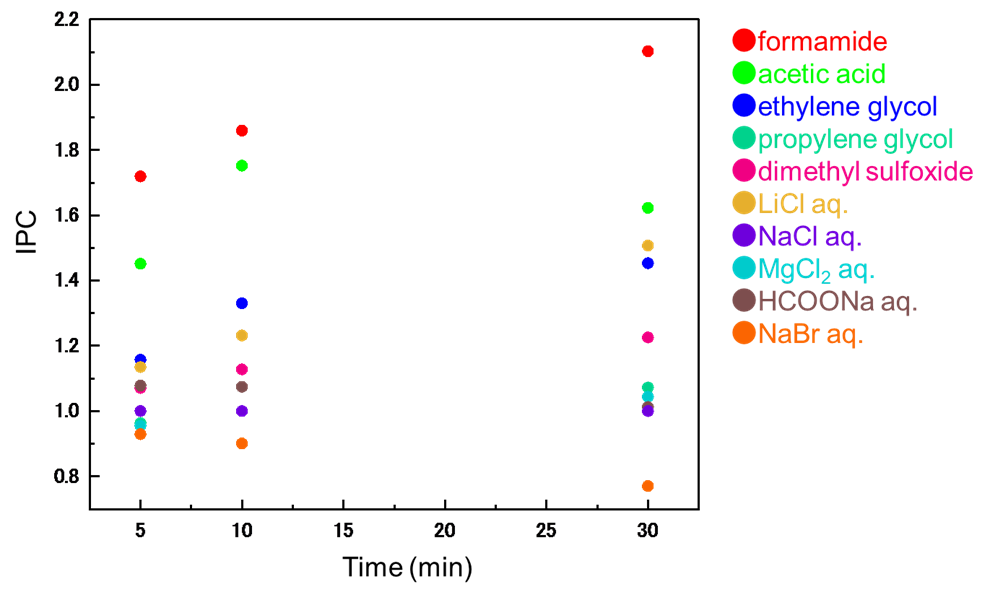


**Figure S3 IPC of pure materials.** This figure shows the IPC of pure materials selected from salt solutions and organic solvents, with five samples each. It can be observed that formamide and formic acid exhibit high IPC. Additionally, there is a tendency for organic solvents to have higher IPC compared to saltwater solutions.

**Table S2 (a) Conductivity, solute density and |pH-7| of NaBr aq. and LiCl aq.^1^ and (b) conductivity of the mixture of PG and salt solutions.**


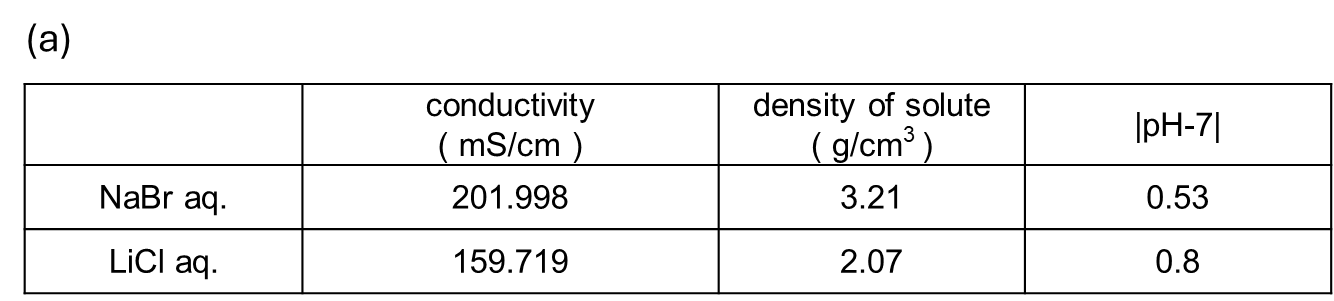


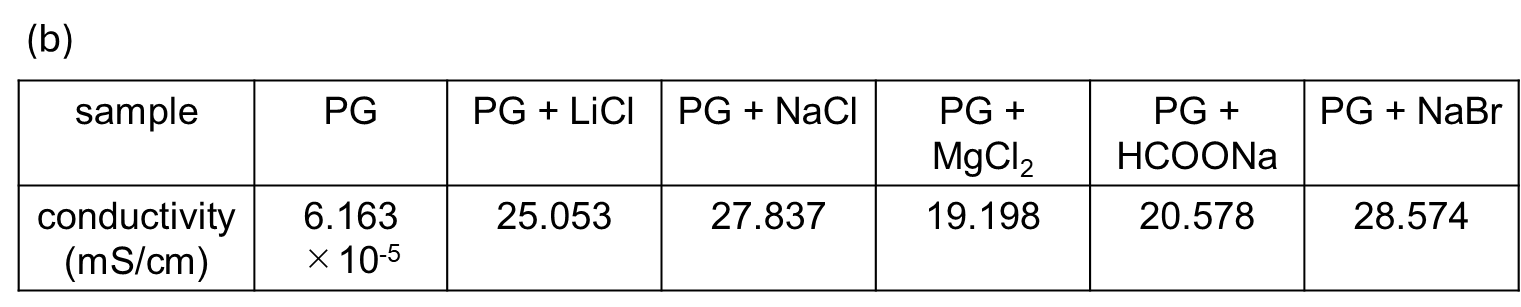


**Table S3 Environmental impact of deicers investigated in this study.**

| Deicer | | Main component | Biochemical oxygen demand (BOD (kg/L)) | Chemical oxygen demand (COD (kg/L)) | Note | Reference |
| --- | --- | --- | --- | --- | --- | --- |
| This work | PG + 25w/w% HCOONa | PG, sodium formate | 0.68 *^a^* | 0.81 *^a^* |  | 2, 3 |
| Salt-based | 25w/w% Clearway SF3 | Sodium formate | 0.0250 *^b^* | 0.0525 *^b^* |  | 4 |
|  | 25w/w% NaCl | Sodium chloride | − | 0.002 *^c^* | Chloride; corrosive effect | 5 |
|  | 25w/w% Snow Tokesuko *^d^* | Calcium chloride | − | − | Chloride; corrosive effect | − |
|  | Tocas Si | Potassium silicate | 0.01 | 0.01 |  | 6 |
| Organic-based | KILFROST DF Plus Type I | PG | 0.961 | 1.38 |  | 2 |
|  | 25w/w% Safe Even When Frozen *^d^* | Urea | − | − |  | − |
|  | Glaco *^d^* | Ethanol | 1.25 *^e^* | 2.08 *^e^* |  | 2 |

*^a^* determined from the average values of PG (BOD: 1.36 kg/L, COD: 1.56 kg/L)^2^ and 25w/w% HCOONa aqueous solution (BOD: ND, COD: 0.0605 kg/L)^3^. *^b^* calculated as 25w/w% aqueous solution. *^c^* value of 23.3% NaCl aqueous solution^5^. *^d^* BOD and COD values of the products are not available. *^e^* values of the main component.

**Table S4 R-squared values for the predicted data using SHAP and the raw data of IPC.**

The R-squared values for both salt solutions and organic solvents, obtained through SHAP, are close to 1 for both predicted and raw data. This confirms the high accuracy of the machine learning model used in this study.

| *R*^2^ | Salt solutions | Organic solvents |
| --- | --- | --- |
| 5 min | 0.9996 | 0.9994 |
| 10 min | 0.9994 | 0.9998 |
| 30 min | 0.9996 | 0.9997 |


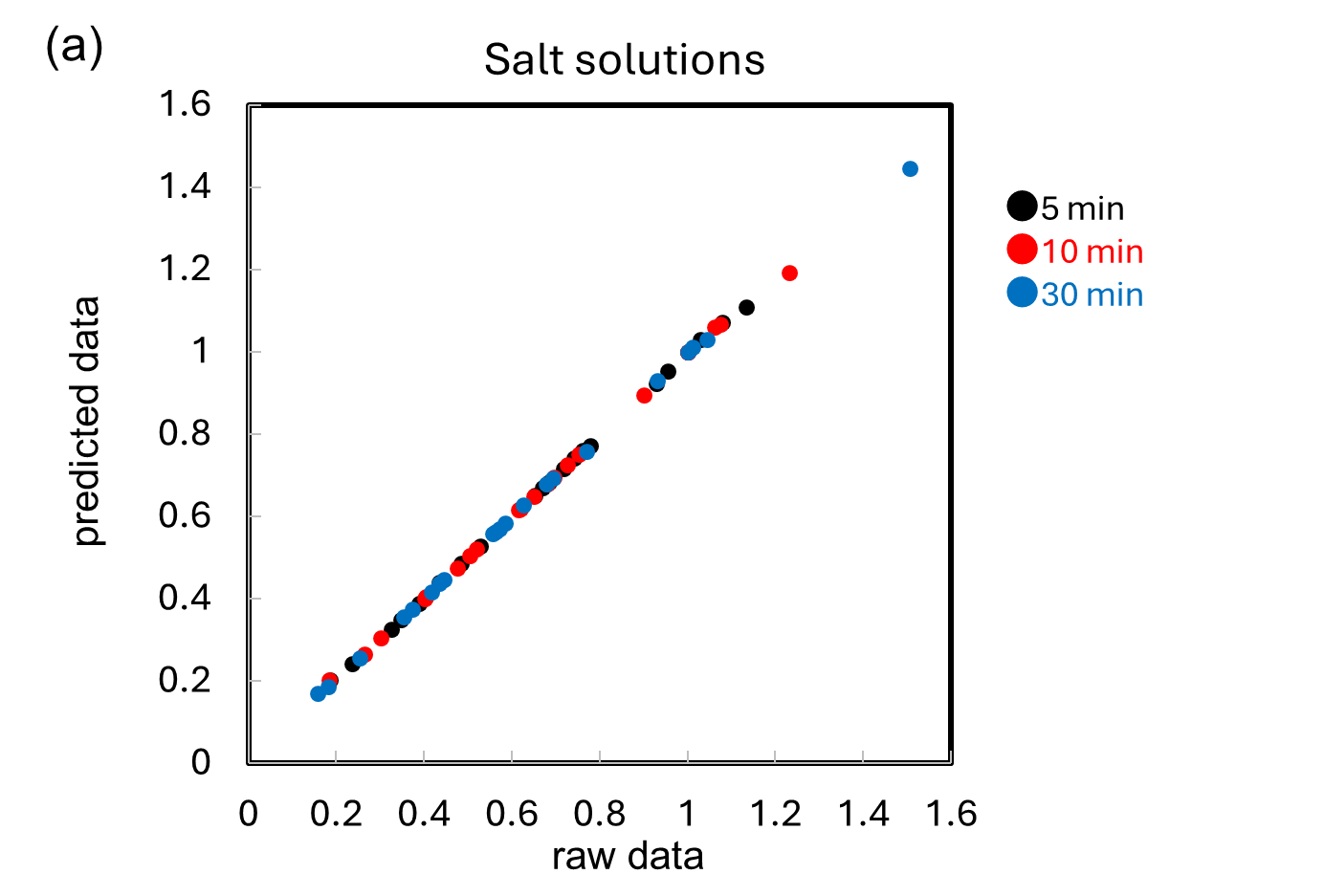


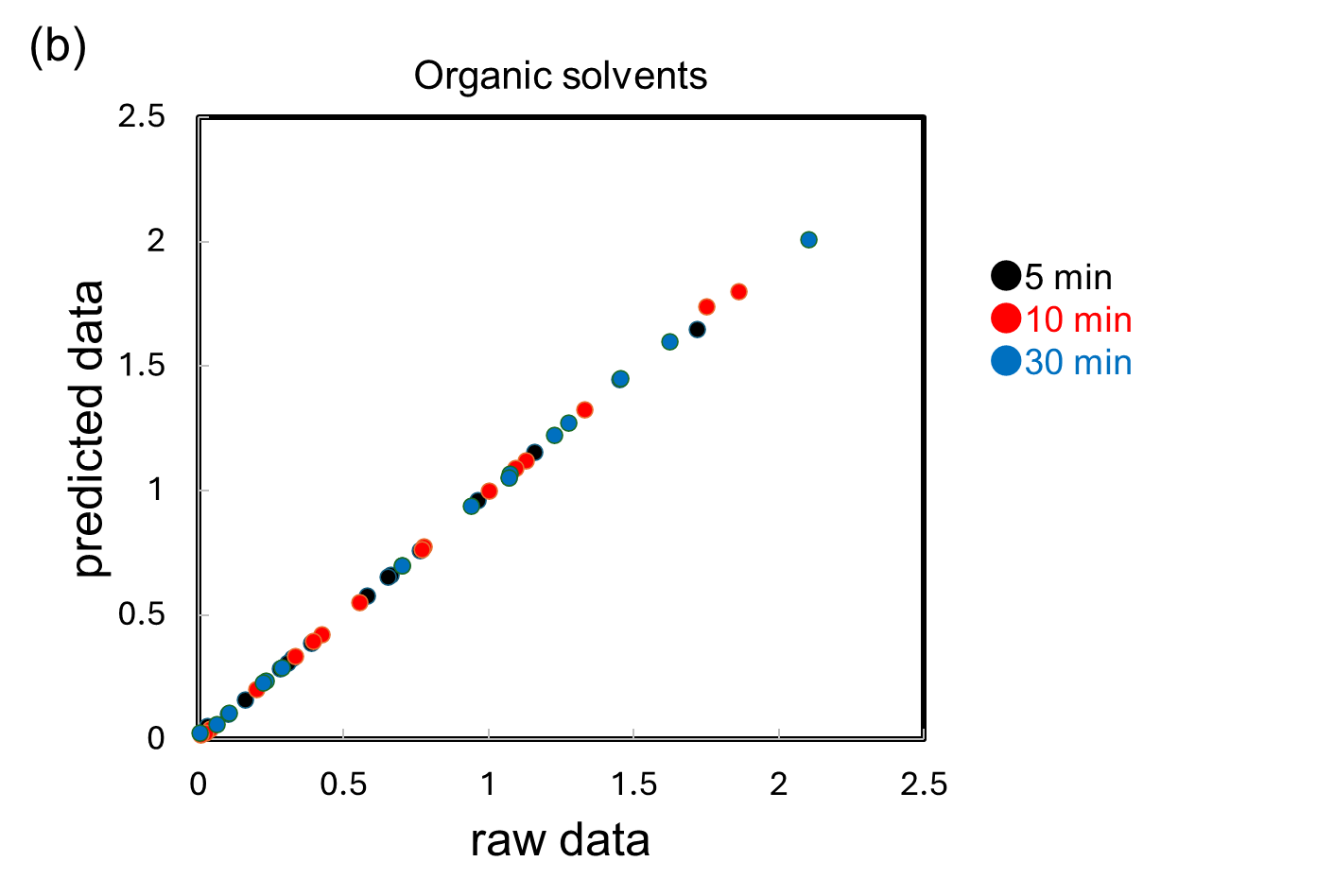


**Figure S4 The relationship between the predicted data using SHAP and the raw data of IPC. (a) salt solutions (b) organic solvents.**

References

1. <https://pubchem.ncbi.nlm.nih.gov/>
2. <https://kilfrost.com/assets/Measuring-environmental-impact--BOD-and-COD-of-heat-transfer-fluids-V5-02042020-(004)_for-web-1609942170.pdf>
3. S. R. Corsi, D. Mericas, G. T. Bowman, Oxygen Demand of Aircraft and Airfield Pavement Deicers and Alternative Freezing Point Depressants, *Water Air Soil Pollut.* **223**, 2447–2461 (2012).
4. <https://productcatalog.eastman.com/tds/ProdDatasheet.aspx?product=71103969&pn=clearway+sf3>
5. X. Li, B. Van Aken, E. Mckenzie, H. Zhang, B. Abboud, W. Davenport, Evaluating the environmental impact of selected chemical de-icers, *Transp. Saf. Environ.* **1**, 220–229 (2019).
6. <https://www.abc-t.co.jp/products/detail/9402.html>
